# Automated Data Annotation for 6-DoF AI-Based Navigation Algorithm Development

**DOI:** 10.3390/jimaging7110236

**Published:** 2021-11-10

**Authors:** Javier Gibran Apud Baca, Thomas Jantos, Mario Theuermann, Mohamed Amin Hamdad, Jan Steinbrener, Stephan Weiss, Alexander Almer, Roland Perko

**Affiliations:** 1 Control of Networked Systems Group, University of Klagenfurt, 9020 Klagenfurt am Wörthersee, Austria; javiergibrap@edu.aau.at (J.G.A.B.); jan.steinbrener@aau.at (J.S.); stephan.weiss@aau.at (S.W.); 2 JOANNEUM RESEARCH Forschungsgesellschaft mbH, DIGITAL, Remote Sensing and Geoinformation, 8010 Graz, Austria; mario.theuermann@joanneum.at (M.T.); alexander.almer@joanneum.at (A.A.); roland.perko@joanneum.at (R.P.); 3 Infineon Technologies Austria AG, 9500 Villach, Austria; MohamedAmin.Hamdad@infineon.com

**Keywords:** 6-DoF relative pose estimation, automated data acquisition, AI-based navigation algorithms, UAS

## Abstract

Accurately estimating the six degree of freedom (6-DoF) pose of objects in images is essential for a variety of applications such as robotics, autonomous driving, and autonomous, AI, and vision-based navigation for unmanned aircraft systems (UAS). Developing such algorithms requires large datasets; however, generating those is tedious as it requires annotating the 6-DoF relative pose of each object of interest present in the image w.r.t. to the camera. Therefore, this work presents a novel approach that automates the data acquisition and annotation process and thus minimizes the annotation effort to the duration of the recording. To maximize the quality of the resulting annotations, we employ an optimization-based approach for determining the extrinsic calibration parameters of the camera. Our approach can handle multiple objects in the scene, automatically providing ground-truth labeling for each object and taking into account occlusion effects between different objects. Moreover, our approach can not only be used to generate data for 6-DoF pose estimation and corresponding 3D-models but can be also extended to automatic dataset generation for object detection, instance segmentation, or volume estimation for any kind of object.

## 1. Introduction

In recent years the popularity of unmanned aircraft systems (UAS) have increased rapidly as they found usage in a wide variety of applications, due to their high mobility, ability to carry multiple sensors, and their low cost [1]. Possible applications include real-time monitoring [2], search-and-rescue operations [3], delivery of goods [4], precision agriculture [5], and infrastructure monitoring (power grids, motorway, rail infrastructure, etc.) [6]. Especially for the latter one, relying on UAS leads to more efficient maintenance processes. In order to achieve these efficiency improvements, the aim of the current developments is to let the UAS perform a completely autonomous flight and recording process. Additionally, this allows the inspection of infrastructure beyond the line of sight of a pilot. We are interested in fully autonomous infrastructure inspection and such an artificial intelligence (AI)-based navigation module allows object-related real-time highly precise navigation of UAS, thus enabling them to follow a predefined recording configuration for the individual piece of infrastructure. However, collecting data of high quality is of utmost importance to ensure the training and validation of such algorithms. While inertial measurement unit (IMU) and global navigation satellite system (GNSS)-based autonomous navigation is well researched and hence can be used to navigate the UAS from its starting point to the infrastructure object, it can not be used to safely navigate in close proximity to the infrastructure object, which is necessary to perform a thorough inspection. The accuracy of GNSS does not allow for high-precision navigation. Moreover, optimal accuracy levels are only achievable in perfect conditions. The interference of GNSS signals with other radio signals or signal reflections caused by the environment are the most common reasons for GNSS distortions [7]. Given the nature of infrastructure objects, e.g., power lines, it is reasonable to assume that a reliable and safe UAS navigation based on GNSS cannot be guaranteed. Therefore, there is a need for new navigation methods that do not solely rely on GNSS signals, while being in close proximity to infrastructure.

One of the first future steps is the development of a novel AI-based pose estimation method given visual information provided by the onboard camera. Given the detection of objects of interest in an image, the method should determine the six degree of freedom (6-DoF) relative pose of each object of interest with respect to the camera. In recent years, deep learning (DL)-based approaches, in particular convolutional neural networks (CNNs), have led to breakthrough performances on many visual tasks [8]. Moreover, the rising availability of powerful graphics processing units (GPUs) for edge devices allows the efficient deployment of neural networks on UAS, thus making it possible to analyze the images in real-time onboard. However, the main drawback of deep learning methods is the requirement for large amounts of data to train and validate these methods. Most of the time, acquiring and labeling data are tedious and time-consuming tasks. Especially for relative pose estimation, where it is necessary to provide relative poses for each object of interest in every single image, it is difficult to collect precise ground-truth data, and thus, the amount of data available for training is limited. Therefore, the main point of this paper is to present a streamlined training dataset generation pipeline that allows the training of AI-algorithms for 6-DoF relative pose estimation for objects of interest. In order to achieve this, we followed two separate strategies in the scope of this work:

– First, a toy example that relies on objects present in the MS COCO dataset [9], as it allows one to focus on algorithm development and testing without spending a long time on data collection and annotation. Most importantly, there exist a wide variety of object detectors trained on MS COCO dataset, and hence, we do not need to train one from scratch, which reduces the annotation effort to the pose of the objects.
– Second, our automated data annotation method that includes object detection in the form of bounding boxes and relative pose calculation consisting of the position and rotation of objects with respect to the camera. The novelty provided by this pipeline is the possibility to generate whole new datasets with custom classes for any kind of objects depending on the target application. Additionally, a detailed 3D-model is created and provided for each object that our pipeline should annotate for. Moreover, this approach is not limited to 6-DoF relative pose annotation but can also be used to generate datasets for object detection, instance segmentation, and volume estimation. In order to achieve accurate ground-truth labeling for these annotation tasks, we had to overcome the challenge of precise extrinsic calibration between camera and motion capture system.

Therefore, in this work we want to illustrate our fully automated data collection process that builds the foundation to train and test novel AI-based navigation modules. Starting with the revision of state-of-the-art methods for automatic data annotation for different visual tasks, we follow up with the detailed description of our method and materials used throughout this work. This includes the two abovementioned strategies. Afterward, example data are analyzed in detail, and their usefulness for the 6-DoF pose estimation and other tasks is reviewed. Finally, we summarize this work and give an outlook on future research.

## 2. Related Work

A key element to AI-based object detection and instance segmentation is a fairly big amount of reliably annotated images. For these applications, annotations involve the bounding boxes that enclose each object of interest in every image and the adequate class tag for each object, among other elements. Satisfying the annotation requirements can be conducted in a manual fashion, meaning that a human annotator must go through each image to manually define the bounding boxes and tags for all the objects present in the image [10,11]. Although still quite common today, it is a major limiting factor due to the extensive labeling efforts for new datasets and applications.

Instead of manual annotations, new techniques perform a semi-automated or fully automated annotation process. A semi-automatic pipeline reduces the human input as much as possible. For instance, the approval or rejection of an annotation is proposed by an algorithm [12,13,14,15,16,17]. In comparison, a fully automatic pipeline totally removes the human interaction. A recent way to achieve this is synthetic data generation and annotation, meaning that data are generated and annotated by an algorithm based on a simulation [18,19,20,21]. This approach offers flexibility and mitigates some of the difficulties of data generation.

Alternatively, researchers explore weakly data annotation, i.e., using noisy annotated data to supervise the labeling of large amounts of data [22]. For example, Zhou et al. [23] took a network trained for image labeling and for each predicted image label they derived an object bounding box with the same label by back-projecting the activated area in the last feature map to the corresponding position in the input image. This way of annotating allows one to greatly cut the time required for the process, but to the extent of our knowledge, its application in more complex tasks is still unexplored.

Object detection and instance segmentation algorithms are the pillars of many modern computer vision applications as they determine the location and totality of an object within an image, allowing one to carry out more complex tasks such as autonomous navigation [24,25], labor automation [26], and security surveillance [27,28].

On top of the already-mentioned applications, the interest on 6-DoF pose estimation in an AI fashion has increased due to the further potential improvement on autonomous navigation and robotic tasks automation [29,30,31]. In this context, 6-DoF pose estimation means approximating the spatial position (X,Y,Z) and orientation (roll, pitch, yaw) of an object with respect to the camera that observes it. Here, 6-DoF manual annotation approaches require an annotator to manually match 3D-model features to the corresponding features on a 2D representation of the same object [32]. These kind of approaches are not only labor intensive but also prone to error as the feature matching may not be accurate enough. A more sophisticated approach extends synthetic data generation and annotation by including the virtual pose of objects and cameras in the annotation [33]. The necessity for a reliable 6-DoF automatic pose annotation pipeline increases as system automation and autonomy increases. This factor motivated us to develop a novel method to automatically annotate a dataset for object detection, instance segmentation, and, most importantly, 6-DoF relative pose estimation. Moreover, the pipeline offers the flexibility to easily integrate new classes to the dataset.

With the introduction of the Benchmark for 6D Object Pose Estimation (BOP) challenge, Hodan et al. [34] unified the 6-DoF pose estimation task by giving an overview of available datasets and summarizing suitable evaluation metrics. Two of the most popular datasets within this challenge are Yale-CMU-Berkeley (YCB)-Video [35] and Linemod(-Occluded) [36,37]. These three datasets offer challenging pose estimation scenarios and a large quantity of data. However, they have two major drawbacks in comparison to our proposed method. First, they only offer a limited distance between camera and object, which is capped at around 100 cm. As shown in Section 3 and Section 4, our method and the accuracy of our motion capture system (MoCap) allow us to record ground-truth poses independent of the distance between object and camera and are only limited to the MoCap’s tracking volume. Second, every dataset included in the BOP challenge places emphasis on how difficult and tedious it is to collect pose data. Providing large datasets, as for example YCB-Video, is only possible with synthetic data, which are generated by placing 3D-models in a virtual space. Due to the ability of automatically annotating data, our approach does not face the problem of generating enough ground-truth data. Other datasets with automated annotation still require postprocessing to come close to the ground truth for their annotation. For example RobotP [38] generates the annotation with structure from motion but still requires iterative pose refinement in order to eliminate estimation errors. By contrast, the accuracy of our MoCap circumvents this problem as is shown later. Therefore, the datasets collected by our pipeline open up new possibilities for future research in 6-DoF pose estimation.

Our implementation generates annotations following the style of the MS COCO dataset [9], a widely used large-scale object detection, segmentation, and captioning dataset that considers 80 object categories such as living animals, food, persons, and transport vehicles, among others. This allows us to easily use our annotations with object detection and segmentation models that were designed to work with MS COCO. Additionally, we enhance the annotation structure by adding the relative object and camera pose as part of the annotation for future pose estimation systems. Summarized, our contributions are:

- An almost completely automated data collection and annotation pipeline for any kind of object and a wide variety of task. For the 6-DoF pose estimation task, we provide the relative pose between objects and camera, the absolute pose of objects and camera with respect to a common world frame, and the corresponding 3D-models. While focusing on 6-DoF pose estimation, our pipeline also provides annotations for object detection and instance segmentation.
- The annotation effort and quality are not limited by the total number of objects present in the image and the distance between camera and objects. Moreover, with our pipeline, we can freely move around the tracking volume and capture whole videos for annotation.
- Overcoming the challenge of exact extrinsic camera calibration for the highest annotation accuracy by defining and solving an optimization problem.

## 3. Materials and Methods

Our automatic data generation and annotation pipeline is divided into three main blocks: 3D-model generation, data acquisition, and data annotation. Besides introducing our pipeline, we also shortly discuss the toy example that we designed to help with development efforts. Moreover, we differentiate between steps that have to be performed only once for the annotation and steps that are always part of our novel pipeline.

**Figure 1:**
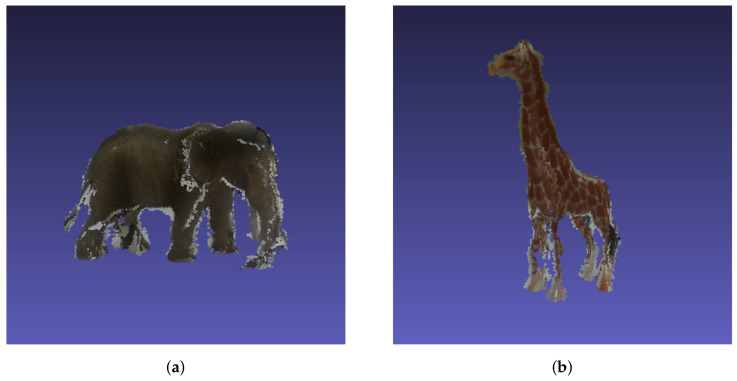
Two example point clouds to visualize the quality of the generated 3D-models. We are able to capture the correct physical dimension of the object and its overall characteristics. The physical extends are 8.2 × 15.3 × 9.0 cm${}^{3}$ and 4.7 × 9.4 × 1.6 cm${}^{3}$ for the elephant and the giraffe respectively. (**a**) Point cloud of our elephant toy figure. (**b**) Point cloud of our giraffe toy figure.

**Figure 2:**
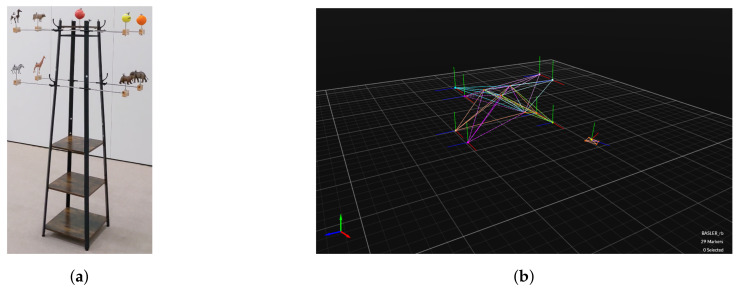
(**a**) Toy Example illustrates the toy example, which is a model of an electric power pole that uses lifelike figures of animals, fruits, and sports equipment, which correspond to 9 distinct labels in the MS COCO dataset. (**b**) Screenshot from MoCap’s tracking software shows the tracked toy example in the MoCap’s tracking software. Best viewed as PDF and in color.

The aim of the 3D-model generation is to capture individual point clouds (PC) that encode the geometry and exact spatial dimension of each arbitrary object to be annotated (*O*). In our case, we used an Intel D435i depth camera [39] and a point cloud stitching algorithm to generate high-quality PCs. For this stitching algorithm, *O* is placed in the center of a plane defined by an unambiguous marker configuration. The depth camera is moved with a fixed distance of 30 cm around *O*, and point clouds are recorded from eight different viewpoints. These recorded point clouds can then be stitched together by simply rotating them to a common plane reference orientation, removing points with a measured depth larger than 33 cm and overlapping them. Subsequently, noise is removed manually from the 3D-model pointcloud with MeshLab [40]. During this postprocessing step, the plane/ground, on which *O* is standing for the recording, is also removed manually to obtain the final clean 3D-model. Exemplary models are showcased in Figure 1, which are part of the toy example described later in this section and can be seen in Figure 2a. There, it is also be argued that we choose toy animals as they are represented by the MS COCO dataset. While the models capture the essence of the animals, there is still some noise left and smaller details, e.g., the elephant’s tail or the giraffe’s legs, are hard to capture due to the low resolution of the camera used. This is not a problem for larger objects. Nonetheless, the models are accurate enough to be used for bounding box and segmentation mask generation in our novel annotation pipeline. The model also captures the physical extent of the objects. These 3D-models have to be generated only once for each object and can then be used in the pipeline to determine an objects position in the image by back projection.

**Figure 3:**
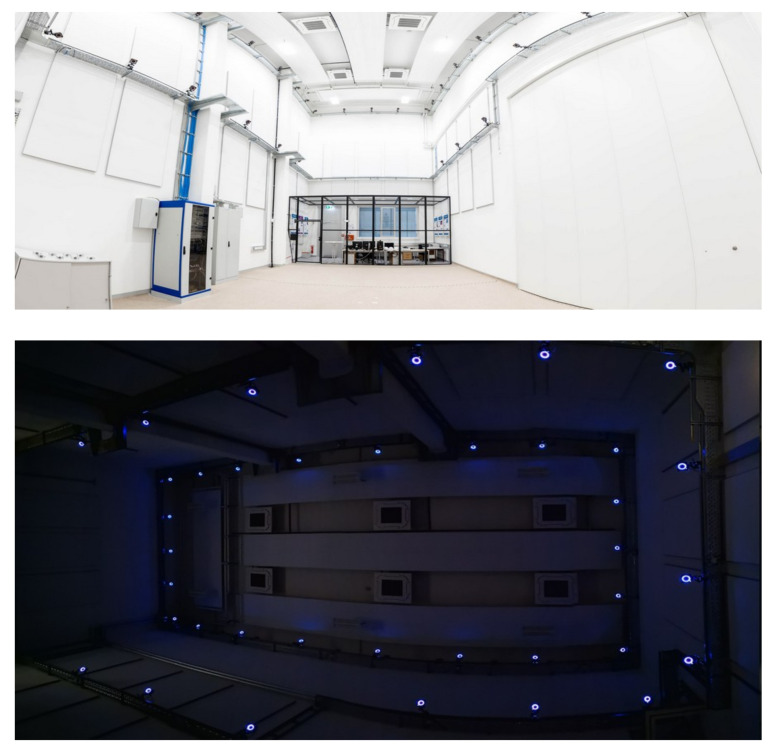
Pictures of our drone hall with the motion capture system used for our pipeline. With a set of 37 cameras we are able to track the hall’s complete volume. The second image is taken upward to capture as many cameras as possible and to showcase the available tracking volume. (**Top**) picture reprinted with permission from Daniel Waschnig. Copyright 2019 Daniel Waschnig. (**Bottom**) picture reprinted with permission from Fred Arneitz. Copyright 2019 Fred Arneitz.

Data acquisition is all about capturing RGB images of the objects of interest along with their 6-DoF pose and the 6-DoF pose of the camera that acquires the frames. This mimics, e.g., the use case of a UAV equipped with a 2D RGB camera performing an inspection task using relative pose estimation to navigate along the structure of interest. The backbone of the data acquisition is our drone hall equipped with OptiTrack, an infrared-vision-based motion capture system (MoCap) that allows one to keep track of the 6-DoF pose of any marked element within submillimeter accuracy with respect to a common reference frame or world origin (W) [41]. In this case, marking an object implies fixing a set of infrared light reflective markers to the object such that MoCap can detect and track it. Our MoCap configuration consists of 37 cameras with a maximal sample rate of 360 Hz, distributed in a 14 × 7 × 10 m volume, granting a flight space close to 100 m${}^{3}$. Pictures of our drone hall and MoCap can be found in Figure 3. Besides the hardware, we rely on a tracking software to compute the ground-truth 6-DoF pose measurement. An example screenshot is displayed in Figure 2b.

As object of interest we introduce a toy example, a collection of objects among lifelike figures of animals, fruits, and a piece of sports equipment, as shown in Figure 2a, which correspond to 9 distinct labels in MS COCO [9]. The main structure of the toy example is defined as a rigid body by fixing a set of markers to it. Then, an individual marker is attached to each object. In this way, we can individually track the pose of each object attached to the main structure.

The basic idea of the toy example is to narrow the efforts to relative pose annotation only, letting us focus on algorithm development and testing. In fact, it allows us to use pretrained, state-of-the-art object detectors, whose output serves as the input for subsequent pose estimation algorithms developed in future research. Moreover, this toy example serves as the evaluation baseline to show our novel pipeline’s annotation accuracy and quality, which is investigated in Section 4.

Although not used during development, the toy example offers the flexibility to keep track of the pose of each *O* even without markers as long as we know the relative transformation between the main structure and each object. In this way, the generated data are not visually polluted by the markers.

Finally, we are annotating by projecting an object’s PC on a virtual plane that emulates the RGB camera, then map the projection to an actual RGB frame. Therefor, camera calibration is mandatory. We perform an intrinsic camera calibration using a checker board and the corresponding calibration tool. The camera intrinsic matrix (*K*) is composed as:

(1) $$K=\left[\begin{array}{ccc} f_{x} & 0 & p_{x}\\ 0 & f_{y} & p_{y}\\ 0 & 0 & 1 \end{array}\right]\;,$$

where $\left(f_{x},f_{y}\right)$ refers to the focal length of both image dimensions, respectively, and $\left(p_{x},p_{y}\right)$ is the principal point on the image plane. Besides using the intrinsic matrix for the projection of PCs into the image, it is also used in a preprocessing step to undistort the recorded images before using them for the annotation.

**Figure 4:**
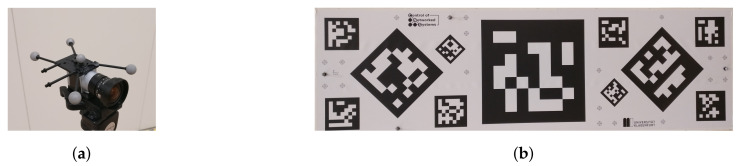
(**a**) shows our RGB camera with markers attached. The marker in the center of the marker constellation defines the position of the camera tracking body in the world frame. In (**b**) one can find the ArUco tag with OptiTrack markers on it used for extrinsic calibration of our camera. Best viewed as PDF and in color.

**Figure 5:**
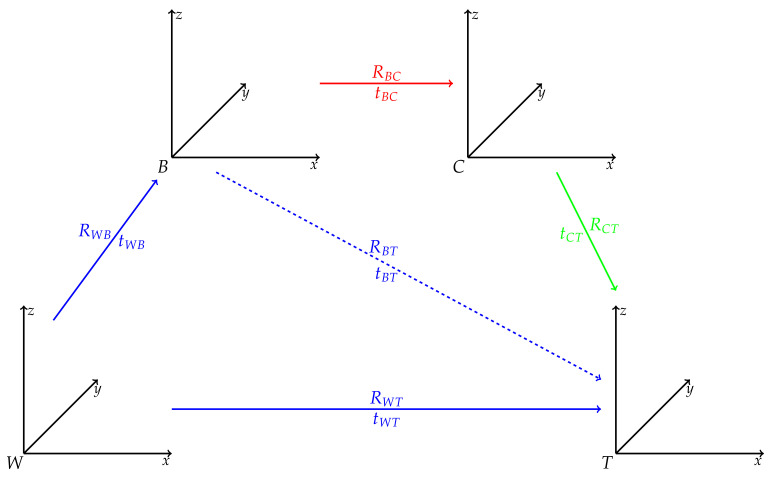
Visualization of the different frames present during extrinsic camera calibration and the transformations between them. The blue-colored transformations can be either directly measured (solid) or calculated (dashed) using our motion capture system. The green-colored transformation can be determined using standard computer vision algorithms, as ArUco tags are commonly used for pose estimation [42]. Finally, the red-colored transformation is the one be determined using calibration. *W* refers to the world frame defined by the motion capture system, *B* is the tracking body frame of the camera, *C* is the camera frame, and *T* represents the frame of the ArUco tag.

Figure 4a shows the camera used, which is a Basler aca2440-35 uc camera paired with a F 2.8/5 mm lens. As can be seen, a set of markers was attached to the camera to track its pose in the world frame (*W*). It is important to note that the pivot point of the camera tracking body (*B*), the marker in the center of the marker constellation, corresponds to the camera’s position in *W* as detected by MoCap and not to the camera plane (*C*). Since the pose of *C* is required, we compute the transformation between *B* and *C* performing an extrinsic calibration. For this, we placed a predefined ArUco tag, shown in Figure 4b, in *W* and attach tracking markers to it. In this way, we could accurately measure the pose of the center of the tag (*T*) with respect to *W*. Additionally, Garrido et al. [42] and Romero-Ramirez et al. [43] proposed algorithms that determine the pose of a tag in an image with respect to the camera frame *C* recording the corresponding image. The relationship between different reference frames in our scenario is illustrated in Figure 5.

It should be noted that a transformation is defined as the rotation $R_{KL}$ and translation $t_{KL}$ of coordinate frame *L* with respect to coordinate frame *K*. Given the MoCap’s current measurement *i*, the position of *T* in *B* can be determined with

(2) $${}_{W}t_{BT}^{i}={R_{WB}^{i}}^{T}\times \left(-t_{WB}^{i}+t_{WT}\right)\;.$$

Please note that $t_{WT}$ is constant for each measurement as we keep the tag fixed and only move the camera. Furthermore, the left subscript *W* highlights that the translation is calculated using the MoCap. Assuming we would know the fixed transformation between *B* and *C* and making use of Garrido’s and Romero-Ramirez’s method, the position of *T* can also be determined for the same measurement *i*

(3) $${}_{C}t_{BT}^{i}=t_{BC}+R_{BC}\times t_{CT}^{i}\;.$$

As before the left subscript *C* indicates that the translation is determined through the current camera image. Based on Equations (2) and (3) an optimization problem is formulated to determine $R_{BC}$ and $t_{BC}$ by minimizing the mean square error for *n* measurements and images as

(4)RBC,tBC=argminRBC,tBC1n∑i=1nCtBTi−WtBTi2(5)=argminRBC,tBC1n∑i=1ntBC+RBC×tCTi−tBTi2 .

Filming the tag board from around n=1500 different angles and positions provided enough measurement points to determine $R_{BC}$ and $t_{BT}$. After optimization, inserting the calculated values for $R_{BC}$ and $t_{BT}$ into the error function yields a value of 0.018. Moreover, the extrinsic calibration to determine the transformation between *B* and *C* has to be performed only once, as long as the camera and marker setup is kept unchanged.

For the data acquisition as such, we set the toy example in an arbitrary location within the hall and recorded the 6-DoF poses of the objects of the toy example, RGB frames of the scene, and the 6-DoF pose of the camera. Overall, the annotation comprises a simulation of the real world. We make the distinction between the virtual world and the real world, as the 3D virtual space in which the simulation occurs and as the space in which the data generation takes place, respectively.

**Figure 6:**
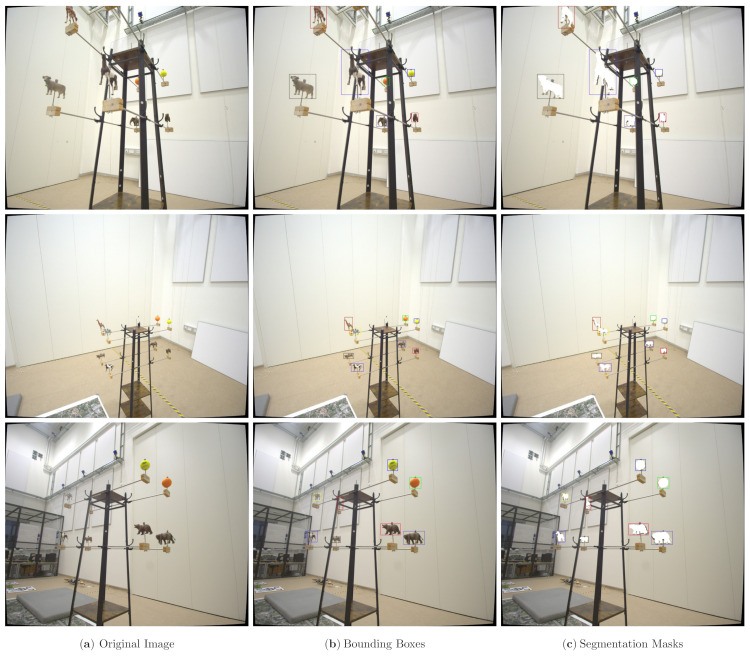
Annotation results produced by our pipeline for the toy example from three different angles. The first column shows the original image, while the second column illustrates the annotated bounding box for each object present in the image. Similarly, the third column displays the corresponding binary masks. The images clearly show that our pipeline is able to produce high-quality annotations sufficient for training and evaluating AI-based algorithms for object detection, instance segmentation, and 6-DoF pose estimation. Only for binary segmentation masks is the annotation not perfect due to the 3D-models not capturing well enough fine details such as the animals’ legs. Best viewed as PDF and in color.

First, the PC corresponding to one of the elements on the toy example was placed in the virtual world in the exact same pose as the corresponding element in the real world. This was possible because MoCap provides the rotation and translation between *W* and *O*. Then, a virtual camera that emulates the same behavior as the real camera *C* was introduced in the same pose as the real camera ($C_{i}$). The PC was projected on the virtual camera plane, the projection transformed to a binary mask, and the enclosing bounding box around the mask was computed. Then, the mask was overlaid on the RGB frame corresponding to $C_{i}$, as shown in Figure 6c.

The projection on the camera plane considers the pinhole camera model. Mathematically speaking this implies the 3D position of each point ($P_{j}$) in a point cloud is projected using the rotation and translation between *W* and *C* depending the camera’s current pose $B_{i}$, which can be defined as the extrinsic transformation ($E_{i}$):

(6)RWCi=RWBi×RBC(7)tWCi=tWBi+RWBi×tBC(8)Ei=RWCiT−RWCiT×tWCi01

**Figure 7:**
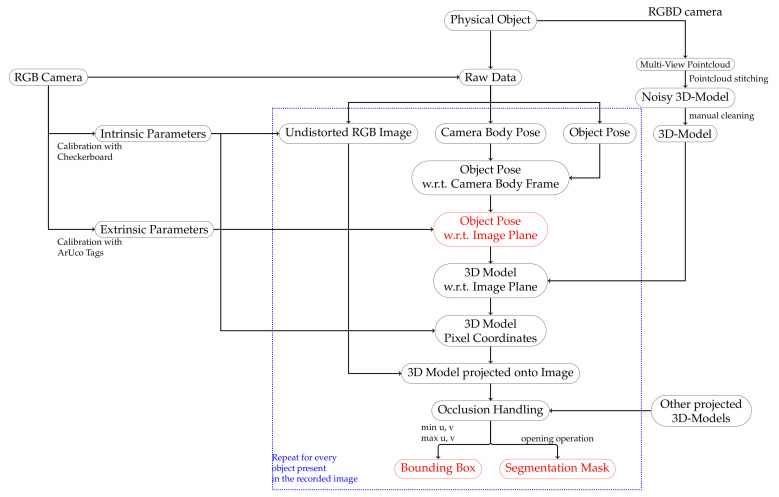
High-level visualization of our data collection and annotation pipeline. For simplicity reasons the pipeline shows the process from the point of view from a single object. However, it should be noted that the part enclosed by the blue rectangle is executed for every frame and then for each object present in the current frame. First, the 6-DoF pose of the object w.r.t., the world frame is mapped to the frame of the image plane, and afterward, using the previously created 3D-model, the pixel coordinates are calculated. The final step consists of occlusion handling and then generating the bounding boxes and binary segmentation masks. On the other hand, every step outside the blue rectangle, namely camera calibration and 3D-model generation, has to be performed only once.

Using the intrinsic matrix *K* as defined in Equation (1), the 2D projection of each point ($p_{j}$) is computed as:

(9) $$\begin{array}{c} p_{j}=K\times E_{i}\times P_{j}\;, \end{array}$$

where points projected outside of the image are simply discarded. With the help of this purely math-based annotation approach and the tracking accuracy of our MoCap, our novel data collection pipeline allows one to freely move around with the camera and capture the objects of interest from different angles in a single recording. Moreover, clean binary masks are generated using a simple opening operation on the binary masks resulting from the PC projection as they tend to have holes. Additionally, we extended the annotation to also take into account occlusion generated by two overlapping objects in an image and removing the occluded object. This can be performed using the current pose of each object and the overlap of the projected bounding boxes. An overview of the data generation and annotation pipeline can be found in Figure 7.

## 4. Results & Discussion

In this section, the annotation results of our data collection pipeline are presented using the toy example introduced in Section 3. Beginning with the evaluation of the pose annotation, we then focus on the bounding box annotation quality. Moreover, we want to evaluate the quality of the bounding box annotation by comparison to manual annotation. The quality is measured in terms of the intersection over union (IoU) score, which indicates how well two bounding boxes overlap [44]. Finally, the quality of the segmentation annotation, a byproduct of our 6-DoF annotation pipeline, is discussed. The reason for lso evaluating the bounding box annotation quality is due to its importance for some 6-DoF pose estimation approaches. These approaches either base their pose estimation algorithms on bounding boxes or regions of interest predicted in a first step [45,46] or train their net to directly also predict the 2D bounding box [29].

The accuracy of our ground-truth 6-DoF pose annotation is completely determined by our MoCap’s accuracy. However, not only the pose annotation but also the binary segmentation mask and the bounding box annotation rely on the accurate measurement of an object’s pose as they are determined through reprojection equations. Besides our MoCap’s accuracy, for the latter two, the annotation quality is also dependent on the correct estimation of the camera’s intrinsic and extrinsic parameters, as well as on the object’s 3D model.

Periodically, our MoCap is calibrated to ensure the highest precision when performing tracking tasks. Especially, before conducting the recordings for this work, we calibrated the MoCap system, and the resulting statistics can be found in Table 1. For every metric, we provide the mean error (ME). The overall reprojection error, averaged over all cameras, measures a single camera’s error when reprojecting a tracker’s position. The error differentiates between the error with respect to the marker’s pose within the 3D tracking volume and the pixels in the image corresponding to the marker. Not only is this metric provided as the average across all camera’s but also for the worst performing camera. The tracking system measures the position of a marker by triangulating the reprojections of each camera in whose field of view the marker is visible. Additionally, a metric is also provided measuring the position error while tracking the calibration wand. Across every metric, it is shown that our MoCap achieves submillimeter accuracy for each marker, and hence, it is possible to state that the annotated position corresponds to the ground-truth position. Moreover, an object in our MoCap system is defined by at least three, fixed markers. Depending on the configuration of the markers, the orientation of the object can be determined by the MoCap. The further apart the markers are, the more accurate the orientation of the object can be determined. Referring back to our toy example and Figure 2, one can see that each MoCap object is defined by a marker on top of the actual object and three additional markers at the top of the pole. By ensuring a sufficient distance between the markers belonging to a MoCap object, we are able to reduce the orientation measurement error to a minimum. Besides that, during a single recording, only the camera is moved, and the toy example stays fixed, which should not introduce movement errors. Moreover, every object’s pose is tracked for several seconds. In combination with keeping the objects fixed, measurement fluctuations are averaged out. Therefore, the accuracy of our MoCap and the experimental setup ensure the true pose of an object is annotated.

**Figure 8:**
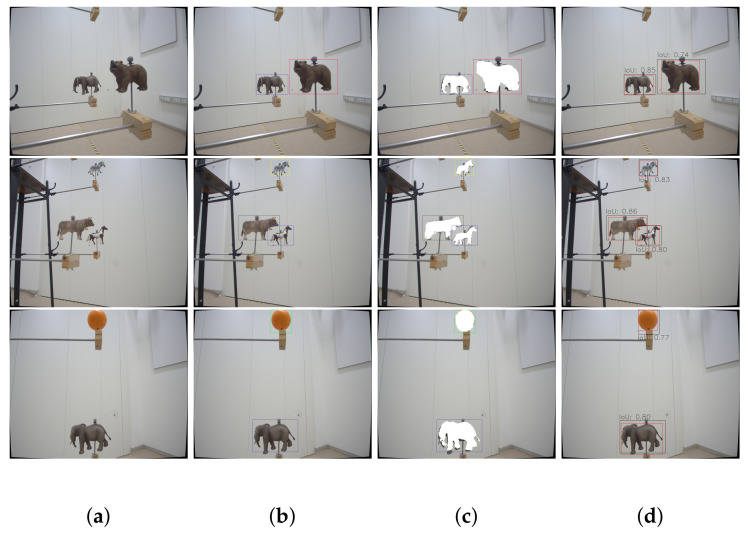
Comparison of our bounding box annotation results to manual annotation. For better visualization, we focus the comparison to close-up recordings of the objects. (**a**) (Original Image) shows the original image, (**b**) (Annotation) the bounding box created by our pipeline, and (**c**) (Binary Masks) additionally includes the corresponding binary masks. Finally, in (**d**) (Comparison), we compare our pipeline’s annotation to the manual annotation, plotted in black and red, respectively. Moreover, we also plot the IoU scores for corresponding bounding box pairs. Best viewed as PDF and in color.

Figure 6 shows example images and the corresponding annotation results generated by our pipeline. Investigating the quality of the bounding box annotation displayed in Figure 6b, one can observe that the bounding boxes are slightly larger than the objects. While evaluating the binary segmentation masks, it can be seen that the projection of the 3D-model does not always perfectly match the object in the image. Therefore, we slightly increase the size of the bounding box to ensure that we capture all details with it. For 6-DoF pose estimation, it is more important to capture the whole object with the help of the bounding box rather than having a tight enclosure of the object. In order to analyze the annotation quality better, we take example images where the camera is closer to the objects and also annotate them manually. The results are presented in Figure 8 with Figure 8d, showing the comparison between our annotation and the manual annotation. On a purely qualitative basis, we can see that the manual annotation produces a much cleaner bounding box. On the other hand, taking a look at the quantitative values, namely IoU scores, for the example data in Figure 8, proves that our annotation is accurate, as the IoU scores never fall below a threshold of 0.74. The average IoU score is 0.79 for the collected toy example data. An IoU score of 0.75 corresponds to the threshold for the strict metrics in the MS COCO challenges [9], and hence, we achieve sufficient annotation quality for subsequent tasks.

**Figure 9:**
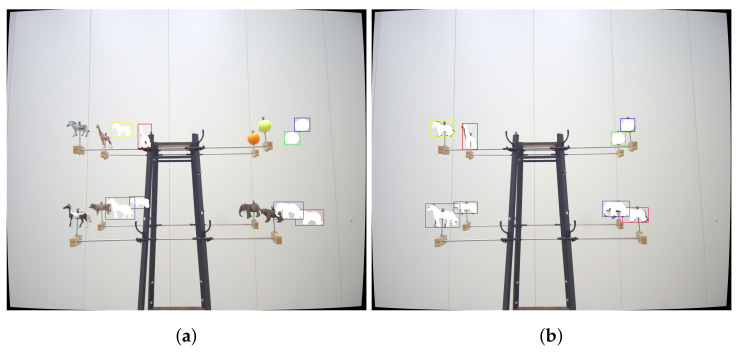
Comparison of the annotation quality for an image annotated using different extrinsic calibrations. (**a**) (Manual extrinsic calibration) shows the annotation result using a manually determined extrinsic calibration between camera image plane and camera tracking rigid body. Given the marker configuration from Figure 4a, we manually measured the transformation from the pivot marker to the assumed position of the image sensor. In (**b**) (Optimized extrinsic calibration), the results are presented for the extrinsic calibration determined by solving the optimization problem. In general, determining the correct extrinsic calibration between rigid body and image plane greatly improves the annotation quality. Best viewed as PDF and in color.

The rather coarse bounding box annotation is due to the projection errors of the 3D-model into the image, which can be best observed in Figure 6c and Figure 8c. Especially for the last row of Figure 8, the influence of the projection error on the annotation quality becomes apparent. Not only do these slight offsets influence the result but also the quality of the 3D-model, as they do not accurately capture small details and the feet close to the ground of the recording plane. Additionally, alignment errors between the recorded 3D-model and the true pose of the object generate mismatches. These alignment errors include, the object being tilted or slightly rotated with respect to the 3D-models defined frame. Besides improving the quality of our 3D-models, the main annotation quality improvement can be achieved by better estimating the camera extrinsics. In Figure 9, the importance of determining the extrinsic calibration by solving an optimization problem is shown. In the beginning, we manually estimated the transformation between the camera rigid body’s pivot point and the assumed image plane, which resulted in the 3D-models being misplaced in the image. After formulating the optimization problem and determining the extrinsic calibration numerically, the annotation quality improved drastically. The manual extrinsic calibration is based on the general assumption in vision-based pose estimation that the transformation between camera body frame (*B*) and camera image plane (*C*) is the identity transformation [R|t]=[I|0]. We slightly adjusted this identity transformation to adjust for the offset caused by the pivot marker along the z-axis (1.5 cm) and a rotation of 180 around the y-axis in the camera body frame, as the camera is mounted upside down to our recording setup. Analysis has shown that even wrong estimations/measurements in the range of 1 mm and 1° significantly influence the projection error. This becomes more apparent the smaller the object is in size. Given the current projection quality, we decided to annotate a bounding box that is slightly larger than the projected 3D-model to ensure that we capture all the details.

**Figure 10:**
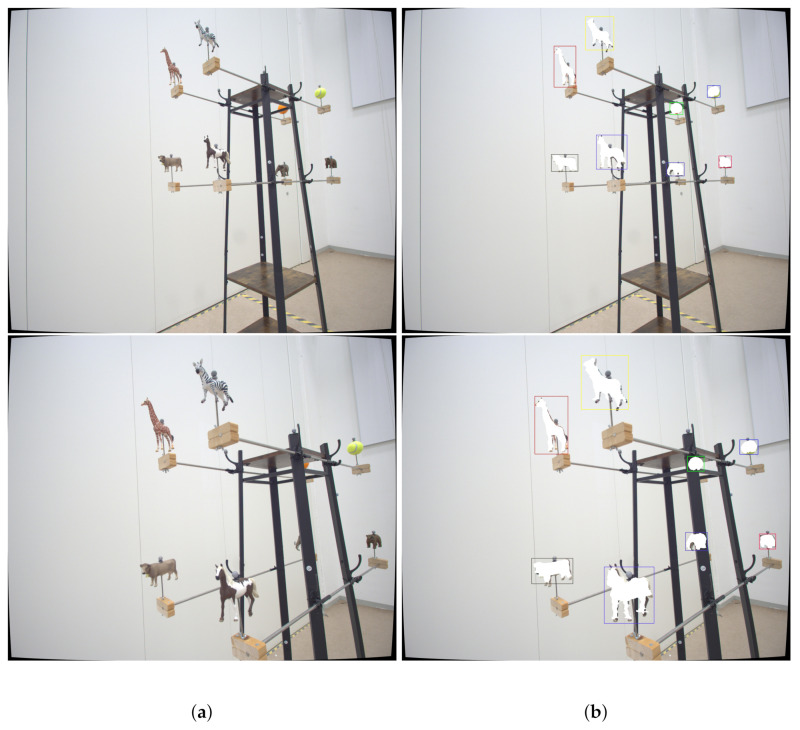
In this figure, we present additional results to highlight the annotation quality of our pipeline. Furthermore, by comparing two similar points of view, it is emphasized that the pipeline’s annotation quality is consistent for different camera distances. In (**a**) (Original Image), we present the original image and in (**b**) (Annotation Result) the annotation results. Best viewed as PDF and in color.

The resulting annotations are of sufficient quality and accuracy to be used for the 6-DoF relative pose estimation task we are working toward. A benefit of our novel approach is that it reduces the annotation time in comparison to manual labeling. Assuming a fast annotator that takes around 10 s per polygon outline per object and for which at least 200 instances per object class are needed, it would take around 16,000 s (roughly 4.5 h) for this annotator to annotate enough data for the previously mentioned toy example consisting of eight objects. Excluding the time necessary for the steps that have to be performed once in advance, namely the 3D-model generation and camera calibration, our pipeline only needs 40 s for the same amount of annotations, if we capture all eight objects in the same frame and if we record with five frames per second while moving around. Even capturing only two or three objects per frame still yields the benefit of reduced annotation time. Additional examples of annotated images are shown in Figure 10.

Besides generating bounding boxes for object detection, collecting position information for pose estimation, and instance segmentation as discussed earlier, there are also other use cases for data generation that our pipeline supports. On the one hand, extending the annotation pipeline by segmentation masks allows for training segmentation networks in addition to object detectors. On the other hand, these masks can also be used for data augmentation and, thus, to artificially increase dataset size. The background in our drone hall is quite static and homogenous. Therefore, the variety of datasets recorded with our pipeline can be extended by cropping out the objects of interest and placing them in images with a suitable background. In addition to generating segmentation masks, our 3D-models also provide the necessary information to determine the object’s volume. Therefore, the annotations generated by the pipeline can be extended to include this information, and hence, our pipeline is also suitable to generate training and evaluation datasets for image-based object volume estimation networks. Moreover, in the case of volume estimation, the additional position and thus distance information provided can be used to help networks better understand ambiguous data points.

Even though the pipeline heavily relies on the tracking technology provided by our drone hall, it can be downscaled to a stereo camera setup. Instead of depending on accurate position measures from a tracking system, a stereo camera setup provides the necessary information to derive an objects pose in one of the camera’s frames. Hence, for each frame, we obtain the relative pose of object with respect to the camera. Moreover, if the position of the camera is known, the global position of the object can also be calculated. Paying attention to the correct orientation of the object’s point cloud with respect to the world frame, the relative pose information is sufficient to project the point cloud into the camera plane once again, thus allowing for the generation of the same annotation as our drone hall approach.

## 5. Conclusions

In summary, in this work, we presented a novel data generation and annotation pipeline for object detection, instance segmentation, and especially 6-DoF pose estimation. For each recorded frame and object present in this frame, the pipeline provides a relative 6-DoF pose with respect to the camera body, a bounding box, and a binary segmentation mask. Additionally, the pipeline can also provide the absolute position of the objects and the camera described in the MoCap’s coordinate system. Finally, the camera intrinsics are also stored in the annotation file, and the objects’ 3D-models are supplied. By optimizing the extrinsic calibration between our MoCap and the camera, we are able to achieve sufficient ground-truth annotation quality, while almost completely automating the annotation effort, thus drastically reducing the time required. Moreover, our design choices lead to a flexible pipeline in terms of creating datasets for any kind of object and a wide variety of tasks. Most importantly, our pipeline will also serve as the foundation for the development of 6-DoF AI-based navigation algorithms in the future.

## Figures and Tables

**Table 1:** Calibration statistics for our MoCap system [41]. For calibration, a wand equipped with markers is used. The exact physical dimension of the wand and marker positions are known to the calibration system. After calibration by moving the wand through the whole trackable volume, the tracking system can calculate the above error metrics based on the exact marker positions and the measured marker positions throughout the calibration procedure. Overall, every metric shows that our MoCap system provides submillimeter accuracy.

Metric	Value
Overall Reprojection (ME)	3D: 0.899 mm/2D: 0.108 pixels
Worst Camera (ME)	3D: 0.766 mm/2D: 0.148 pixels
Triangulation (Residual ME)	0.9 mm (Recommended 3.0 mm)
Overall Wand Error (ME)	0.252 mm

## Data Availability

Not applicable.

## Abbreviations

The following abbreviations are used in this manuscript:

6-DoF	six degree of freedom
UAS	unmanned aircraft systems
AI	artificial intelligence
IMU	inertial measurement unit
GNSS	global navigation satellite system
DL	deep Learning
CNN	convolutional nerual network
GPU	graphical processing unit
MS COCO	Microsoft common objects in context
BOP	benchmark for 6D object pose estimation
3D	three dimensional
YCB	Yale-CMU-Barkley dataset
MoCap	motion capture system
PC	point cloud
O	object
RGB	red green blue
W	world origin
K	camera intrinsic matrix
B	body frame
C	camera plane
T	center of tag
R	rotation matrix
t	translation
IoU	intersection over union
ME	mean error
